# O8.1. EXAMINING RELATIONSHIPS BETWEEN PSYCHOTIC EXPERIENCES AND SUICIDAL IDEATION IN ADOLESCENTS USING A NETWORK APPROACH

**DOI:** 10.1093/schbul/sby015.237

**Published:** 2018-04-01

**Authors:** Daniel Nunez, Andrés Fresno, Claudia van Borkulo, Philippe Courtet, Víctor Arias, Viviana Garrido, Johanna Wigman

**Affiliations:** 1 Universidad de Talca, Chile; 2 University of Amsterdam; 3 CHU Montpellier, University of Montpellier; 4 Universidad Católica del Maule; 5 University of Groningen, University Medical Center Groningen

## Abstract

**Background:**

Suicide is the second cause one of the leading causes of death in young individuals. Timely and adequate identification of individuals with suicidal ideation could prevent from suicidal behavior. Psychotic experiences (PE) have been shown to increase levels of suicidal ideation (SI) in the general population. Therefore, detailed investigation of the relationship of PE and SI is relevant. However, the exact nature of the relationship between these two phenomena remains unclear, which is intensely debated nowadays. Given both the high complexity of SI and behavior and the fact that its expression has a trans-diagnostic nature, a fruitful approach to gain new insights about its relationships with psychiatric symptoms might be the application of network analysis, which could be helpful to elucidate specific associations existing between PE and SI.

**Methods:**

A specific type of network analysis, the Ising model, was used to examine connections between dichotomized questions on psychotic experiences and suicidal ideation in a cross-sectional study with 1685 adolescents from the general population aged 13–18 years. To assess psychotic experiences, we used an item generation deductive method (Hinkin, 1995) of two pre-existing scales that we adapted in previous studies: the Brief Self-report Questionnaire for Screening Putative Pre-psychotic States (BQSPS; Liu et al., 2013), and the Community Assessment of Psychic Experiences—Positive scale (CAPE-P15; Capra et al., 2013). The questionnaire encompassed 15 items addressing the following dimensions: perceptual anomalies (PA; 3 items); bizarre experiences (BE; 6 items); social anxiety (SA; 3 items); and negative symptoms (NS; 3 items). Suicidal ideation (SI) was assessed by six items of the Columbia–Suicide Severity Rating Scale (C-SSRS; Posner et al., 2011), adapted for being used as a self-report questionnaire. Severity of SI was rated on a 6-point ordinal scale in which 1 = wish to be dead, 2 = nonspecific active suicidal thoughts, 3 = thoughts about how to commit suicide, 4 = suicidal thoughts and intentions, 5 = suicidal thought with detailed plan, and 6 = intentions to conduct plan.

**Results:**

SI was mostly connected to the PE domains perceptual anomalies (PA) and bizarre experiences (BE), which have higher strength values in the network. Central nodes within these domains, as indexed by higher centrality measures (strength and betweenness) were: auditory experiences (PA1: hearing voices when you are alone), persecutory ideation (BE1: feelings of being persecuted; BE2: conspiracy against you), and social anxiety (SA) (SA1: I cannot get close to people).

**Discussion:**

Through a network analytic approach, our results add new insights to previous findings concerning the associations between psychotic experiences and suicidal ideation, suggesting that perceptual anomalies (mainly auditory experiences), social anxiety (being distant to people), and bizarre experiences (paranoid beliefs) are connected in a meaningful way to suicidal ideation in a network of symptoms in a sample of non-help-seeking adolescents. Given the potential advantages of the network analysis to study psychopathology and suicidal behavior, its usage can contribute to a better understanding of the nature of the complex relationships between these phenomena. Future network analysis studies should include additional symptom domains to analyze whether the associations between PE and suicidal behavior are undifferentiated; are specifically and independently associated, regardless of antecedents of mental disorders; or if the associations are not specific, but merely reflect a higher underlying risk of suicidal behavior as a function of psychiatric symptoms or mental distress.

